# Ingestive Behavior and Precision Nutrition: Part of the Puzzle

**DOI:** 10.1016/j.advnut.2025.100531

**Published:** 2025-10-01

**Authors:** Annabel Biruete, Pius Sarfo Buobu, Robert V Considine, Erisa Met Hoxha, Heather A Eicher-Miller, Kimberly P Kinzig, Anita A Panjwani, Cordelia A Running, Giorgia Rutigliani, Dennis A Savaiano, Amanda Veile, Patricia G Wolf, Richard D Mattes

**Affiliations:** 1 Department of Nutrition Science, Purdue University, West Lafayette, IN, United States; 2 Indiana University School of Medicine (IUSM), Division of Endocrinology, Indianapolis, IN, United States; 3 Department of Psychological Sciences, Purdue University, West Lafayette, IN, United States; 4 Department of Anthropology, Purdue University, West Lafayette, IN, United States

**Keywords:** human, precision nutrition, ingestive behavior, culture, sensory, appetite, food intake, gut–brain axis, microbiome, diet

## Abstract

The marked individual variability in response to common dietary exposures necessitates tailoring of dietary guidance to individuals, or small groups of individuals with similar needs, to optimize health. This is a complex task requiring integration of environmental, cultural, psychological, and biological contributions. Work in the area of precision nutrition is an effort to translate science into practice. A research roadmap developed through a National Institutes of Health conference identified many of the inputs that require quantification. Better characterization of ingestive behaviors is one key area. The aim of this narrative review is to summarize current understanding of the influences of age, sex, body mass index, ethnicity, and genetics on ingestive behaviors, including culture, sensory function, appetite, dietary intake, the gut–brain axis, and microbiome. To do so, the extant literature was accessed through search engines relevant to the various topics covered. Outcomes assessed varied topically. In addition to compiling evidence on the nature and magnitude of these relationships, this review highlights the degree of individual variability in attributes or responses to an intervention. More broadly, it documents: *1*) that cause-and-effect relationships are difficult to establish as most are dynamic and interactive; *2*) there are inherent and learned contributions to both behavior and biology that will require different considerations and offer different opportunities for manipulation; *3*) a focus on intuitive approaches may not be as successful as desired; *4*) that external influences can, and often do, override internal influences of biology; and *5*) there are multiple ways to construct healthful diets. At the same time, it is vital that improved methods to characterize the multiple relevant inputs to ingestive behavior be developed. It is hoped that the evidence compiled here will inform efforts to develop precision nutrition guidance.

## Introduction

Increasingly, policy-makers and healthcare providers seek dietary recommendations to optimize health and prevent and/or treat chronic disease in individuals or small groups with common needs. The NIH “Precision Nutrition Initiative,” part of its 2020–2030 strategic plan, aims to understand and implement strategies based on personal biological traits, lifestyle practices, and environmental considerations. Such an effort aims to improve the quality and duration of life. However, many questions related to individual dietary “optimization” remain to be answered. First and perhaps most relevant: Is there an ideal diet for a single or small subgroup of individuals? Furthermore, what are the variations in this ideal diet that would still maintain optimal health? Additionally, what human adaptations might occur that would diminish or enhance this "ideal diet" over time? Solutions will require integration of a wide array of dynamic inputs and accommodate ongoing inevitable variability at the diet–health interface.

Understanding ingestive behavior, the integrated processes of food seeking, selection, and consumption, is essential to developing effective and individualized dietary strategies within precision nutrition frameworks. This was highlighted at a workshop titled “Precision Nutrition Research Gaps and Opportunities.” It was supported by the National Heart, Lung, and Blood Institute, National Institute of Diabetes and Digestive and Kidney Diseases, and NIH Office of Disease Prevention and was held on 11–12 Jan, 2021. The meeting comprised scientists with varied expertise who explored the multiple factors that influence physiological responses to varying dietary approaches and how artificial intelligence and deep-learning techniques could contribute to the goal of preventing or treating disease and optimizing health. To fully grasp the complexity of these behaviors, we believe it is helpful to consider how they have been shaped over time by evolutionary pressures and environmental changes in human nutrition and metabolism.

Archaeological evidence suggests that regular meat consumption began in the human lineage ∼2 million years ago [1], with growing reliance on butchery and mechanical processing methods (e.g., pounding and grinding) [[1], [2], [3]]. Between 1.8 and 0.4 million years ago, our ancestors adopted cooking, which degraded plant fibers and denatured meat proteins—reducing the costs of mastication and digestion, increasing energy/nutrient yield, and altering gut microbiota—ultimately enhancing nutrient absorption and metabolic efficiency [[4], [5], [6]]. These innovations contributed to hallmark human traits, including small teeth and jaws, short guts, and large brains [7], as well as behaviors such as divisions of labor, ecological flexibility, and diversified foraging practices [8,9].

*Homo sapiens* appeared on the fossil record around 200,000 y ago and were hunter-gatherers for most of their history; searching for food in their local environments and migrating seasonally to better hunting and foraging grounds [10]. Some scientists propose that this historical dietary pattern provides the best evidence for what an optimal diet might be [11]. The evidence to support this hypothesis is limited by time and is dependent on difficult research approaches. The potential for nutritional deficiencies in hunter-gatherer diets remains, especially in marginal environments [12]. The emergence of agriculture, starting some 10,000 y ago, marked the onset of a major transition in dietary habits for many populations, as people became more sedentary, tending crops and domesticating animals, which in turn limited seasonal migration [13,14]. The suggested “improvement” in dietary patterns during the agricultural period has been questioned, with limited data supporting improved diet quality and nutritional health [13,14].

The industrial revolution of the 18th and 19th centuries brought dietary habits to a third major crossroad, with transportation and storage of foods essential for the new urban workers who purchased their food in the marketplace. This industrial age of food procurement brought with it significant dietary deficiency conditions such as beriberi, scurvy, and pellagra [15]. These “defects” of the modern food system have been mitigated primarily through improved transportation and processing technologies including fortification strategies, but there is growing awareness of new threats to long-term health associated with unbalanced or over-nutrition, such as obesity, cardiovascular and other systemic diseases, diabetes, cancer, and others that are partially due to poor diets. The 3 unique food systems in the history of humankind had considerable variability due to changes in climate, natural resources, and geography. What adaptations did humans evolve to support this wide variety of dietary practices? Consider the Arctic Inuit and the Massai pastoralists of Kenya and Tanzania, 2 different yet successful groups who depended on very different climates, environments, food opportunities, and thus diets. These examples illustrate how human food-seeking and consumption behaviors have evolved in response to diverse environmental pressures, laying the foundation for modern variability in ingestive behavior.

As knowledge of past and evolving human genetics improves, it is becoming increasingly clear that whereas much of the human genetic code is constitutive, there is also considerable capability to adapt to environmental factors. Single-nucleotide polymorphisms (SNPs), occurring only a few thousand years ago, have made approximately one-fourth of humans “lactose tolerant” due to changes in promoter activity of the lactase gene throughout adult life [16]. Accumulating evidence also supports a coevolution of taste function and diet [[17], [18], [19]]. There are many more examples of genetic changes, driven by evolutionary pressures, that have affected dietary practices and health. Furthermore, there is only preliminary understanding of the even more rapid changes in epigenetic outcomes that likely allow for adaptations within lifespans or with immediate offspring. Does an ideal diet depend on recent epigenetic changes? What are these changes, and how might they influence precision nutrition efforts? Taken together, these genetic and epigenetic examples reinforce the idea that ingestive behavior is shaped by both long-term evolutionary adaptations and shorter-term biological responses. Recognizing this variability is key to designing precision nutrition strategies.

Food seeking, food choice, and consumption are essential prerequisite behaviors that provide the energy and nutrient substrates to support reproduction, growth, and health. Thus, they are critical elements for the understanding and implementation of precision nutrition. This was explicitly acknowledged in the NIH precision nutrition workshop report on research gaps and opportunities in the field [20]. That report also emphasized the importance of contributions by personal traits such as age, sex, BMI, ethnicity, and genetics to ingestive behaviors, terms that require clear definition to be adequately addressed.

### Sex and gender

Sex is a biological category, fundamentally defined by the type of gametes an individual produces (ova or sperm) and associated with female (XX) or male (XY) sex chromosomes, respectively [21]. A very small number of individuals exhibit chromosomal variations in addition to XX and XY (e.g., Turner and Klinefelter Syndromes) [22]. In practice, sex is assigned at birth based on external reproductive anatomy (genitalia). Genetics, gonads, and genitalia—collectively known as the “3G sex”—are usually aligned and internally consistent, though natural variations occur [23]. Gender is multidimensional and can be defined as “culturally contextualized social and structural experiences as well as expressions of identity” [24]. Gender encompasses the social roles, norms, and institutional structures that shape how individuals experience and express their identity in relation to sexual phenotypes [24,25]. It is not only individually expressed but also socially constructed, and maintained through relationships, cultural roles and expectations, and power dynamics [26]. According to the Endocrine Society, “Sex is an important biological variable that must be considered in the design and analysis of human and animal research. The terms sex and gender should not be used interchangeably” [27]. However, most biomedical studies do not distinguish or specifically define sex and gender [28]. Although we hope the definitions provided can help promote greater clarity and consistency surrounding these terms, this review inevitably draws upon studies reflecting these broader inconsistencies.

### Body size and BMI

Body size refers to the overall physical dimensions and composition of a person’s body. It includes factors such as height, weight, muscle mass, fat mass, bone structure, and body proportions. Body size can vary greatly between individuals due to genetics, age, sex, nutrition, and physical activity. BMI is a widely used, inexpensive, and noninvasive method for assessing a dimension of body size, specifically, body weight in relation to height. It is calculated by dividing a person’s weight in kilograms by the square of their height in meters. This calculation yields a numerical value used to classify individuals into 4 general categories: underweight (BMI <18.5), normal or healthy weight (BMI between 18.5 and 24.9), overweight (BMI between 25 and 29.9), and obese (BMI of 30 or higher) [29]. BMI is a generally useful population-level health indicator, as high BMI is associated with elevated risks for metabolic conditions such as type 2 diabetes, hypertension, and cardiovascular disease [30]. BMI also has important limitations [31,32]. It does not adequately differentiate between fat and lean muscle mass, so this measure can misclassify muscular individuals as overweight or obese. It can also fail to account for population differences in body proportions, bone structure, and fat distribution. Therefore, in the context of precision nutrition and healthcare, BMI should not be used in isolation. Individual health assessments should include additional indicators such as body fat percentage, waist-to-hip ratio, metabolic biomarkers, and physical fitness [32]. In combination with these other metrics, BMI serves as a starting point for evaluating health, while allowing for more nuanced and individualized interpretations [32,33].

### Race and ethnicity

Race and ethnicity are often used interchangeably, but they refer to different concepts. Race categories are based on perceived physical traits, whereas ethnicity relates to shared cultural or national identity [34,35]. Both race and ethnicity are *socially* constructed categories that vary cross-culturally. Still, they are *biologically* relevant to health; as such, they are frequently used in biomedical research. Importantly, “self-identified race and ethnicity commonly correlate with geographical ancestry and, in turn, geographical ancestry is a contributing factor to human genomic variation [36]. Although self-identified race and ethnicity correlate with the frequency of particular genomic variants at a population level, they cannot be used exclusively to predict an individual’s genotype or drug response [36].”

Race and ethnicity are, therefore, potentially useful but imprecise tools in health and nutrition research. Like sex and gender, they are often self-reported, used interchangeably, and poorly defined, which can reinforce the mistaken belief that health disparities are rooted *only* in genes and biology [35]. This ignores social factors (racism and discrimination, limited healthcare access, provider bias) that also strongly influence health outcomes [37]. Precision medicine and nutrition offer more refined ways to study health that take these into account along with a multitude of biological, social, and individual factors.

### Culture and society

Culture can be defined as a shared system of knowledge, beliefs, practices, and normative behaviors that are transmitted socially between individuals within a population [38]. Culture shapes individuals’ perceptions and interpretations of their social world, influencing behavior through internalized understandings rather than external structures. Society refers to the structured network of relationships and institutions that organize human interactions [38]. This encompasses roles, norms, and institutional arrangements that guide behavior and maintain social order. Society, in this sense, is the macro-level structure within which individuals and groups operate. Although society and culture are distinct, they are deeply interrelated. Societal structures provide the framework within which cultural meanings are produced and transmitted, whereas culture imbues these structures with significance, guiding behavior and social interaction.

**FIGURE 1:**
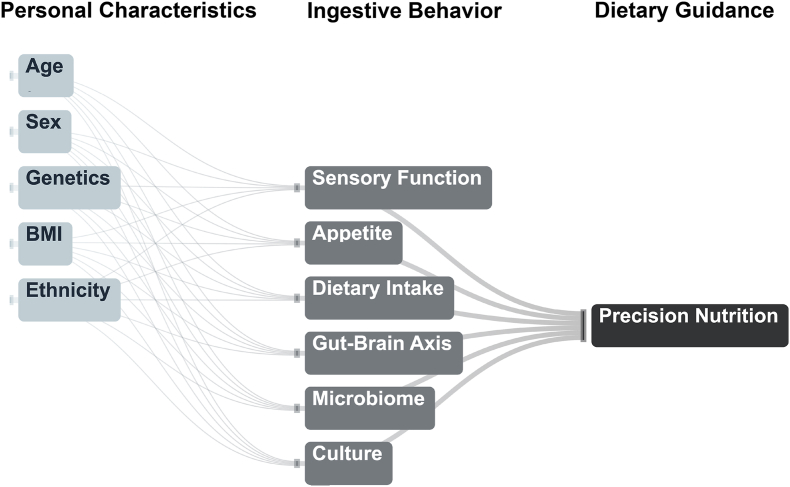
Flow diagram of the format used to explore the influences of contributors to dimensions of ingestive behavior and their relationship to precision nutrition.

Building on the historical and biological context described previously, this narrative review aims to examine how individual variability in key traits, including culture, appetite, sensory function, dietary intake, the brain–gut axis, and the microbiome, affects ingestive behavior and dietary needs. The goal is to support the development of precision nutrition strategies by highlighting the magnitude and relevance of this variability and its implications for personalized dietary guidance. For clarification, Figure 1 depicts the approach taken in this review.

Each of the topics addressed below has its own unique set of metrics, approaches of study, and relationship to ingestive behavior. Consequently, it is not possible to use a single, common rubric to review the evidence across sections. A narrative review approach was adopted to permit greater flexibility for inclusion of varied forms of evidence that enabled a broader and more conceptual assessment of the extant literature.

### Cultural factors

#### Search and strategy

Integrating cultural context into precision nutrition is essential for developing strategies that are equitable, effective, and grounded in real-world complexity. This section provides an overview interdisciplinary scholarship on the associations between culture, food, and health. Sources span nutrition science, anthropology, human biology, public health, epidemiology, and broader social and basic sciences. The selection strategy prioritized geographic, economic, ethnographic, and sociopolitical breadth, enabling a comparative approach and supporting alignment of precision nutrition with actual dietary practices. This section provides a framework for understanding human dietary experiences that is expansive enough to reflect the diversity of human experiences, yet flexible enough to be tailored to culturally meaningful practices in application.

The search focused on literature published since 2000, emphasizing review articles with a global scope and from the past decade, to ensure inclusion of current, generalizable knowledge. Over half the sources are review articles offering theoretical or conceptual syntheses; ∼40% are original research articles in scholarly (mainly scientific) peer-reviewed journals; the remainder are editorials, public health recommendations, and book chapters. Roughly half of the original research articles are quantitative, whereas half utilize qualitative and/or mixed methods. Collectively, these sources provide a multidimensional evidence base to guide future research and inform culturally attuned precision nutrition policy and practice.

#### Introduction

Food holds significant cultural meaning in human societies. In addition to its biological function, food acts as a fundamental marker of individual social identity [39]. Cultural factors shape population and individual variation in dietary intake, simultaneously influencing the types of foods people eat, attitudes toward eating, and the social context in which food consumption occurs [40]. These influences manifest through various norms, beliefs, and practices that vary across regions, communities, and even within households and across age/sex groups [41].

Cultural norms dictate meal timing, frequency, and portion sizes, which all affect energy and nutrient intake [42]. Furthermore, different cultures have unique preferences for specific *types* of foods that are often tied to historical, economic, and geographic contexts [43]. Food also plays a critical role in the formation and maintenance of social bonds [44]. The capacity to obtain sufficient, culturally appropriate food through socially accepted means enables individuals to perform their social roles and engage in meaningful social interactions, including daily activities and communal events [45,46].

Here, we briefly review cultural influences on individual variability in dietary intake. Necessarily, we use a biocultural approach; the scientific study of how human biology and culture interact, and how culture affects human biological capacities and constraints. Although beyond the scope of this review, it is important to note the variety of subsistence behaviors practiced in contemporary human societies. These range from (but are not limited to) household-level food production (hunting and gathering, foraging-farming, fishing, pastoralism, small-scale farming) and market-based and mixed economies (cash-cropping, markets and mixed subsistence economies), and highly delocalized, industrialized, food production and distribution systems [10,47]. In wealthy and industrialized settings, food choices can be vast and plentiful, though potentially limited by socioeconomic barriers [48]. In a broader context, dietary intake and status, ultimately, are shaped by a complex web of cultural, social, economic, environmental, political, and global forces [49,50].

#### Age/life-stage

Culture impacts dietary intake at every stage of life, from childhood to old age, shaping not only what and how people eat, but also their broader relationship with food. Age-related changes in appetite, health, and social circumstances are filtered through cultural norms, which influence individual food choices and overall nutrition.

#### Infancy

Energetic demands are high in infancy, a period of rapid brain and body growth and immune system maturation. Caregiver culture plays a crucial role in shaping infant food consumption. The WHO and American Academy for Pediatrics recommend exclusive breastfeeding for the first 6 mo, followed by continued breastfeeding in conjunction with complementary foods for 2 y or beyond [51]. Although lactation is an evolved strategy [52,53], breastfeeding practices (timing, frequency, duration) are heavily influenced by cultural norms, maternal education, economic factors, social infrastructure, and family dynamics [52,54]. In some societies, public and extended breastfeeding is normalized, whereas in others, public breastfeeding is stigmatized, and workplace policies can limit its continuation beyond early infancy [55,56]. Similarly, the decision to use formula or and/or complementary foods, and selection and preparation of foods, are shaped by social circumstances, financial conditions, caregiver education, exposure to infant food marketing, and infant-specific needs and factors [[57], [58], [59], [60], [61]]. Cultural practices vary, with some promoting breastfeeding for a set period or introducing solid foods early, whereas others encourage delayed transitions [53,62].

#### Childhood

Children require nutrient-dense foods for body growth, cognitive development, and immune function. Cultural beliefs and practices shape how caregivers introduce foods and establish eating habits [63]. Caregivers, through cultural norms, determine the types of food their children eat, portion sizes, and the frequency of meals, shaping children’s preferences [64,65]. In many cultures, “alloparents” (nonparent caregiving helpers such as grandparents, aunts and uncles, cousins, older siblings, and family friends) play important roles in feeding young children [[66], [67], [68]]. In others, parents are the primary caregivers and decision-makers [69,70]. Cultural practices involving food as a reward or punishment can influence children's appetite and intake patterns. For example, in some Westernized and industrialized cultures, sweets and snacks may be used as a reward or during special holidays, promoting a preference for sweet, energy-dense foods [71].

In addition to family environments [72], important food-related learning occurs with media exposures and in health care, educational, and formalized child-care settings [47,49,61]. When caregivers and teachers eat the same foods alongside young children, it encourages healthy eating habits and creates a positive mealtime atmosphere [71,72]. However, for teachers and/or students from ethnic minority cultures, differing food norms can lead to tensions with institutional expectations [[73], [74], [75]]. Additionally, cultural differences, whether meals are eaten at school, at home, or brought from home, can influence food quality, eating patterns, and exposure to different dietary norms [76]. School lunch programs, which are widespread in many countries, provide affordable or free nutritious meals; efforts to provide culturally relevant and desirable meals are variable and ongoing [[77], [78], [79]].

#### Adolescence

Adolescents require nutrient-dense foods to support growth and pubertal development. Cultural ideals around body image significantly impact their eating behaviors in interaction with socioeconomic constraints [80,81]. In cultures that idealize thinness, adolescents (especially females) may restrict dietary intake through dieting or fasting, whereas in cultures that value larger body sizes, dietary intake may increase to align with those ideals [82]. Peer influences, advertising, and social media further shape adolescent eating habits, potentially promoting either unhealthy consumption and/or dieting trends [83,84]. As adolescents gain independence, they begin making their own food choices [81]. These choices are shaped by their cultural and family background, and whether they maintain family traditions and/or cultural staples (especially in immigrants), or adopt new habits [80,85,86]. Eating meals outside the home can become more common during adolescence, influenced by social dynamics and increased autonomy [81]. At the same time, schools continue to shape dietary decisions through nutrition education, food offerings, and meal programs [77]. By providing low-cost or free meals, schools can help offset malnutrition among low-income students and, optimally, promote healthy eating for all students [87].

#### Adulthood

Cultural norms regarding work, family, and food variably influence dietary intake in different settings [88]. For instance, in cultures where long working hours are common, adults may rely more on meals optimizing convenience, often leading to higher energy intake [89]. Irregular and overnight work hours (shift work), in particular, negatively impacts eating patterns with consequences for metabolic health [90,91]. In contrast, cultures and contexts that emphasize family mealtimes and home-cooked food often promote healthier food choices and portion control [92]. In lower-income settings (especially rural), the timing and nature of adult dietary intake are often linked to cultural factors and socioeconomic constraints related to household resources and composition [41,93].

#### Pregnancy and lactation

Biological demands, cultural norms, and socioeconomic factors shape dietary intake during pregnancy and lactation, influencing both maternal health and infant outcomes. These stages increase energy, protein, and micronutrient needs (e.g., iron, calcium, folate) to support fetal growth, milk production, and maternal well-being [94]. Many societies normalize changes in maternal diets to meet these needs, encouraging beneficial foods while avoiding others (e.g., taboos) that are believed to be deleterious [[95], [96], [97], [98], [99]]. Pregnancy often alters appetite, with manifestations of cravings and aversions that may shape maternal diets to some extent [100]. Cultural beliefs, and even medical recommendations, about healthy eating in pregnancy and optimal gestational weight gain, are also variable across populations, communities, and health systems [101,102]. Additionally, postpartum recovery diets, such as confinement foods, can emphasize nutrient-dense meals to promote healing and milk production, though in some studies, postnatal diets may be restrictive with little nutrient diversity [103,104]. Family and community support, including meal provisioning, is common in many societies and also impacts postpartum dietary intake; those with limited support may struggle to prepare meals and consume healthy foods [105].

#### Aging

Cultural beliefs about aging shape eating patterns in later life. In societies where elders are valued and welcomed in communal meal settings, they tend to maintain balanced diets [106]. Conversely, in cultures associating aging with decline, reduced social interaction may lead to decreased appetite and dietary intake [107]. Health conditions like diabetes or osteoporosis often require dietary changes, which can be constrained by socioeconomic and other structural factors [95], particularly in low-income settings [108]. Retirement also influences intake; cultures that view retirement as rest may see reduced energy consumption, whereas active-aging societies promote healthy eating through physical and social activities [109]. Living arrangements play a role, as older adults who live with their families generally have more stable diets, whereas those in nursing homes or living alone may experience declines due to limited food access or social isolation [107]. Reduced energy consumption can result in reduced nutrient intake leading to deficiencies in essential nutrients—especially those that are less well absorbed or produced in lower quantities in elderly individuals (e.g., B12) [110].

#### Sex and gender

Biological nutritional needs vary by sex and by reproductive status (see pregnancy and lactation, above). However, gendered differences in eating norms and behaviors—independent from reproductive status—are also prevalent across human societies. Cultural expectations and realities shape food preferences, access, and behaviors, creating variation in individual dietary intake based on sex and gender [111]. For example, certain foods may be classified as “masculine” or “feminine,” influencing eating patterns and varying across cultures [112]. Furthermore, nonbinary individuals can encounter challenges with food choices due to noninclusive binary-based social norms [113].

Body ideals (e.g., for fatness, thinness, muscularity) also affect food choices. Culturally shaped beliefs and pressures may influence healthy or disrupted eating practices; these can manifest in gender-specific ways [114,115]. Gendered food practices can also reflect social and household power dynamics [112]. In some cultures, males receive larger portions or more food autonomy, whereas females, traditionally managing caregiving and cooking, may prioritize family needs over their own intake [41]. However, in many family-centered societies, communal meals promote balanced access to food within households [93]. Religious traditions can also impose dietary practices by gender. For instance, fasting practices may be gender-specific during specific rituals; other religious beliefs may affect eating behaviors during menstruation [116]

#### Genetics

Genetic and cultural history shape contemporary variations in dietary intake by influencing how populations respond to different foods [117]. For example, people from certain African, Middle Eastern, and European regions with lactase persistence consume dairy easily, whereas those who lack this adaptation may avoid or limit dairy due to lactose intolerance [16,118]. Similarly, historically agriculturalist populations with more amylase alpha 1 gene copies, metabolize starch efficiently, making them more accustomed to carbohydrate-rich foods [119]. In contrast, Arctic populations, with specific polymorphic variants in genes related to lipid and carbohydrate metabolism, may struggle with blood sugar regulation when exposed to modernized high-starch diets [120]. As such, dietary intake is not solely based on preference, but also on how an individual's biology interacts with certain foods, highlighting the importance of tailored nutrition.

#### BMI

Culture shapes individual dietary intake and influences BMI through eating habits, food preferences, and social norms [121]. Some cultures promote small, frequent meals, whereas others encourage large portions or feasts, leading to variations in energy consumption [122]. Traditional foods also play a role; diets with lower nutrient density tend to increase BMI, whereas nutrient-dense diets help maintain lower BMI [123,124]. For example, several traditional and culturally significant foods from the Peruvian Andes—purple corn, legumes, and quinoa, have a variety of antiobesity properties [[125], [126], [127]]. Religious or cultural food restrictions, like fasting or vegetarianism, also affect food choices and weight outcomes [116]. As noted previously (gender section), cultural ideals surrounding body type ideals can affect individual food consumption practices [114,115]. Culture also interacts with economic factors to shape dietary norms, sometimes promoting and normalizing high BMIs [128,129]. For example, convenience foods, which are frequently rich in fats, sugars, and refined carbohydrates, can become dietary staples where they are relatively inexpensive and widely accessible [129,130]. Even when more affordable and nutritionally dense staples such as starchy vegetables, grains, and legumes are available, they may be seen as impractical or undesirable due to the time, cooking knowledge, and resources required for preparation [130,131]. Additionally, influences like predatory marketing and lack of access to nutritious foods (food “deserts” and “swamps”) can disproportionately affect low-income communities [[132], [133], [134], [135]]. In contrast, cultures and contexts that support and prioritize fresh, home-cooked meals help individuals manage healthier weights [136,137]. These cultural influences show that dietary intake patterns are shaped not only by individual preference, but also by beliefs surrounding food and nutrition and socioeconomic constraints, resulting in significant variations in BMI.

#### Race and ethnicity

Racialized and ethnic minority groups are often excluded from mainstream health campaigns that prioritize dominant populations. An example is the promotion of dairy consumption in the United States, despite widespread lactose nonpersistence among several non-European populations, that have been encouraged to adopt dietary norms misaligned with their cultural traditions and/or biological needs [138]. Foods associated with marginalized communities are also frequently devalued or labeled as low status, whereas the same foods may gain prestige when adopted by dominant cultural groups [[139], [140], [141]]. In the context of globalization and migration, traditional foodways can become powerful symbols of cultural resilience and identity, as seen in the growing global visibility of Yucatec Maya cuisine through culinary tourism, restaurants, and the sale of cookbooks far beyond Yucatán [142,143]. Still, when “ethnic” or “superfoods”—such as Peruvian quinoa, a grain, or Laotian kaipen, a green algae—are commodified for global markets, this visibility can paradoxically undermine the livelihoods and food security of small-scale producers in their communities of origin [144,145].

#### Summary

Precision nutrition holds transformative potential for improving health outcomes, but its success depends on grounding tailored dietary guidance in the lived realities of diverse populations. This section has highlighted the central role of culture in shaping dietary intake across life stages, sex and gender, race and ethnicity, and genetic and body composition profiles. Although precision nutrition aims to deliver more targeted interventions, its effectiveness will be shaped by the broader familial, cultural, economic, and political contexts in which people live. These contexts are inseparable from human biology and evolved histories. Without supportive structures such as access to affordable, culturally appropriate foods, community-based health knowledge, and policies that reduce nutritional inequality, even the most finely tuned recommendations may fall short. Precision nutrition must therefore embrace cultural and socioeconomic complexity as fundamental design principles. By integrating localized cultural contexts with human biology, scientists can develop individualized nutrition strategies that are scientifically rigorous, socially relevant, and impactful.

### Appetite

#### Search and strategy

To inform this section, an emphasis was placed on original research identified through searches of online databases via search terms of appetite, hunger, fullness, desire to each, prospective consumption, thirst, food intake, energy intake, and linking them to age, sex, gender, genetics race, ethnicity, and BMI. Relevant citations identified in the original articles, as well as other reviews and meta-analyses, were also considered.

#### Introduction

Appetitive sensations are experienced by all people on a continuous, but constantly fluctuating basis. They are widely believed to motivate the initiation and termination of eating events and thus contribute to eating frequency and portion size, respectively. In turn, eating frequency and portion size determine total energy consumption, so appetitive sensations are assumed to be predictive of energy intake. There is some evidence for a meaningful correlation, but that analysis noted marked individual variability of responses. Even under the clearest experimental conditions (i.e., 4.5-h postingestive responses), appetite ratings accounted for only ∼25% of the variance in energy intake. Of course, energy intake is also modulated by multiple inputs besides appetite (e.g., cultural expectations for eating all one is served or not), which leads to additional variability and weakening of the association. Thus, documentation of a reliable relationship has proven to be elusive. This is highlighted by a review of 462 published papers that revealed a significant association in only 49.7% of reports [146]. This lack of a robust association may reflect a weak biological relationship, fail to capture a substantive contribution due to inadequate measurement of appetitive sensations and/or energy intake, or be obscured by high intra- and interindividual variability. Mean responses in study subgroups have been suggested to yield limited insights, as this metric masks individual differences in appetitive responsiveness, and possible distinct phenotypic groups that display more consistent responses to various interventions (e.g., diet, exercise) [147,148]. Accumulating evidence supports this view, but raises new questions about the behavioral and biological implications of such distinctions [149].

#### Appetite and energy intake

The literature is replete with evidence for contributions of environmental, behavioral, and physiological controls of both appetite and dietary intake. Each plays a variable role under different circumstances. For example, stronger correlations between hunger and dietary intake are noted during the work week compared with weekends [150]. Moreover, this phenomenon may vary with different appetitive sensations; that is, hunger may be a stronger (albeit still weak in absolute magnitude) driver of meal initiation (eating frequency) whereas environmental factors (e.g., social norms, and food availability) may be stronger determinants of eating termination (portion size). Additionally, the directionality of the underlying hypothesis regarding appetite and energy intake is unclear. If the strength of appetitive sensations reliably varies interindividually, as some data suggest [149,151], and appetite is really a dominant driver of intake, then clear associations should be apparent. However, stable high hunger may promote high energy intake or reflect a low level of intake with antithetical implications for body weight; high in the former case and low in the latter. Similarly, chronic low hunger could prompt low intake or result from high intake. Bimodal relationships would therefore be expected to obscure associations, but recent data have failed to identify bimodal distributions [149]. Overall, more current evidence indicates that appetitive sensations are only weakly associated with energy intake [146,149,[152], [153], [154]].

#### Assessment and measurement error

The most common approach to measurement of appetitive sensations is by self-report to a series of questions about attributes such as hunger, fullness, desire to eat, prospective consumption, and thirst. Assessments of the reliability of these ratings have yielded mixed results. Reported coefficients of repeatability (2 X Std Dev) vary from 24 to 30 mm (on a 100 mm visual analog scale) under fasting conditions and 23–29 mm after a fixed meal when successive ratings were obtained on the same day [155]. When ratings were collected under similar conditions over 2 successive days, reliability ratings were ∼29–52 mm under fasting conditions and 14–38 mm after a standard meal [156]. When measuring hunger and fullness as means over waking hours during days, the coefficients of reliability of individuals reporting low, medium, and high daily hunger or fullness range from 10 to 16 mm [149]. The reliability of shorter (1 wk) and longer-term (17 wk) interindividual ratings is strong, with correlations ranging from 0.67 to 0.88. Correlations are comparable between hunger and fullness with stronger correlations for thirst [149]. Mean daily variance between individuals is markedly greater than the mean variance within individuals [149,151]. Within-subject values range from 3% to 7% whereas between-subject values range from 93% to 97% of the variance across sensations.

The importance of assessing individual variability of responses, in contrast to group means, was highlighted by an NIH Workshop exploring the biological controls of appetite [157] as well as others [148,158]. Tables for computing study power for paired and unpaired design trials to explore this topic have been published [155]. However, it is important to note that testing conditions will influence variability. For example, response formats (e.g., paper and pencil compared with electronic recording) yield data with different variance [147]. Additionally, variability is reduced for postprandial ratings compared with fasting or preingestive ratings [149,159], though this too, is variable.

#### Age

Assessment of appetitive sensation variability associated with aging is complicated by the higher prevalence of chronic diseases and medications used to manage them in older adults. Polypharmacy is prevalent and can be associated with weight gain [160] or weight loss [161]; both can have adverse health consequences. As the mixture of drugs used and effects on appetite will be idiosyncratic, it is most instructive to consider age-related variability in appetite among healthy individuals. A meta-analysis of studies of such individuals revealed that after overnight fasting, hunger was 25% ± 24% (∼16 ± 13 mm on a 100 mm scale) lower in older than younger adults [162]. In a postprandial state, hunger was 39% ± 30% lower (∼15 ± 11 mm). Fullness was 37% ± 73% (∼8 ± 13 mm) greater after an overnight fast in older than younger adults. No significant differences were noted for fullness postprandially. However, intervention trials exploring appetitive ratings after consumption of a single meal in healthy participants across the lifecycle have been inconsistent. Some work indicates the variability in appetitive responses is similar in younger and older individuals [163], whereas other evidence indicates the dynamic range is compressed in older individuals [164]. This apparent discrepancy may be attributable to the wider age range of participants tested in the latter study; children may report more extreme ratings. Mean appetitive sensations across days do not differ by age [165].

#### Sex and gender

Mean appetitive sensation ratings are generally comparable for females and males [165], especially preprandially [166,167]. Some data suggest selected (inconsistent across studies) postingestive appetitive sensation ratings change more in females than males [163,166,168], but the differences are not always significant after correction for daily energy intake [168]. There is also a suggestion that females show slightly more rapid changes in sensation ratings. Some data suggest females have greater variability in selected responses {e.g., coefficient of repeatability values for fasting [54.5 mm (females) compared with 33.6 mm (males)] and intrameal hunger [66.6 mm (females) compared with 34.7 mm (males)]}, but not for other outcomes such as postprandial AUC or 10 h mean hunger [169]. However, such data are based on small sample sizes, leaving open questions of generalizability. Others report coefficients of repeatability for appetitive sensation ratings are more uniformly comparable between the sexes [163,168].

#### Genetics

A genetic basis for individual variability in appetitive sensations has been established in infants, children, and adults [[170], [171], [172], [173]]. Early analyses of dietary recalls suggested 32% and 19% of the variance in premeal and postmeal hunger/fullness ratings had a heritable component, and 39% of the variance of change of hunger/fullness over the course of a meal was genetically determined [170]. Whether these findings reflect a heritable influence on number usage or actual sensation is uncertain.

Later, findings from several large twin studies with children confirmed and extended these observations. One study with 5435 8–11 y old twin pairs reported 63% of the variance in satiety and 75% of the variance in food cue reactivity was attributable to genetics, with 21% and 16% due to shared environment and 10% and 15% to nonshared environmental influences for satiety and food cue responsiveness, respectively [174]. A contributor may be a heritable influence (62%) on eating rate [175]. Children deemed “food-responsive” had higher eating frequency, and those labeled “less-satiety-responsive” reportedly consumed larger portions [176]. Effects on eating frequency were smaller at younger ages when eating is largely under parental control, and then increase with age. Another trial of 87,782 twin pairs from 0.5 to 19.5 y of age reported the genetic contribution increased with age from ∼42% to ∼75% in 19 y olds [177]. Values were largely similar in different global geographic areas and between sexes.

Subsequent work has indicated that these relationships are also present in adults [178]. Some work has suggested that fat mass and obesity-associated gene (FTO), the FTO obesity risk genotype, may exert part of its effect through modulation of appetitive sensations [179]. A small meal trial contrasting responses of AA and TT homozygotes suggested they have comparable fasting hunger and fullness sensations, but the former have an attenuated hunger response [180]. However, a later comparable study failed to confirm this observation [151]. There is also a suggestion of a greater effect with high protein exposure [181], but this requires replication. Although there is a heritable influence on appetitive sensations, evidence linking this to BMI suggests the effect is small [172,182,183]. One analysis indicated a composite genetic risk score for variants of the FTO gene accounts for ∼3% of the variance in BMI in children and adults [126]. Though other work suggests this estimate may be high, as combined, SNPs of the FTO, MC4R, and LEP (leptin gene) genes accounted for only 2.1% of the variation in log (BMI) in Black South Africans [184]. To date, studies attempting to link a specific gene to food cue reactivity have been largely negative or mixed (e.g., significant effects in females, but not males or differences between studies) [[185], [186], [187]].

#### BMI

Studies of populations representing a range of BMI values generally reveal nonsignificant associations between BMI and appetitive sensations [163]. Coefficients of repeatability for hunger and fullness among individuals with and without obesity ranged from 3.24 to 3.37. Similarly, contrasts between individuals who are lean or have obesity indicate the groups do not differ in fasting sensation ratings nor in responses to various interventions (e.g., macronutrient content, physical food form; single meal compared with over days) [159,165,[188], [189], [190], [191]]. Additionally, individuals classified as obesity prone or obesity resistant did not differ in hunger or satiety responses to under or overfeeding [192]. Coefficients of repeatability were nearly identical between groups and conditions. Under or overfeeding in these subgroups on 1 d resulted in changes in mean appetitive sensations the next day, but had little effect on the variability of responses. Attempts to identify SNPs in a range of genes associated with appetite in clinical populations have largely failed to identify significant effects [193]. There are studies of SNPs in genes associated with a variety of neural circuits linked to appetite, but the outcomes of these trials generally focus on adiposity indices, and there is only an inference that this is mediated through appetitive sensations.

#### Race and ethnicity

Data related to ethnicity effects on appetitive ratings are sparse and have been assessed under highly disparate conditions. Nevertheless, the findings consistently reveal no marked differences. For example, comparable ratings were observed in trials comparing ambient temperature effects on appetite in Han and Hui adults [194]; monosodium glutamate exposure effects among Malay, Chinese, and Indian 9–11 y old children for fasting or postprandial responses [195]; exercise effects on South Asian compared with White European males [196] and African American compared with White adults [197]; or between African American and White adults following meals varying in glycemic index [198,199]. Exemplary coefficients of reliability of African American and White females for hunger, fullness, prospective consumption, and desire to eat are ∼35 under control conditions and following 2 levels of exercise [151].

#### Summary

Taken together, there is marked intra- and interindividual variability in appetitive responses. Interindividual variability is greater and stable over time. The preponderance of evidence indicates that appetitive ratings are more similar than dissimilar in groups based on age, sex, genetics, BMI, or ethnicity. The association between appetitive ratings and energy intake or BMI is weak. The degree to which this is due to limitations in the measurement of appetite or the contributions of numerous other drivers of these outcomes is not established. Until this latter issue is resolved, the best approach to addressing appetitive sensations in guiding ingestive behavior is uncertain.

### Oral sensory experience

#### Search and strategy

PubMed and Web of Science were used to search for original research articles and reviews using sensory terms (flavor, taste, smell, odor, chemosensation, oral sensation) and the factors of interest (age, gender/sex, genetics, race/ethnicity, and BMI). Recent reviews were pulled and citation lists and new citations examined for new relevant work. Emphasis was placed on articles that examined outcomes related to ingestive behavior, rather than just fundamental differences in these factors.

#### Introduction

The sensory experiences of taste and smell play a crucial role in food choice and, consequently, in nutrition and overall health. Although extensive documentation exists on the variability in sensory function and overall sensory experiences of foods [200,201], the influence of this variability on diet quality and subsequent health outcomes remains a subject of debate. Several studies have demonstrated associations between sensory impairments and various health issues [193]. These findings underscore the importance of intact sensory function for maintaining adequate nutrition and overall health. However, although the impact of sensory dysfunction is well-established, the contributions of normal, healthy sensory variability to nutrition are less clear, or at least less consistent. Furthermore, chemosensory experience and preferences may change overtime [150,[202], [203], [204], [205]], showing plasticity, and the degree to which these changes are driven by factors such as aging, dietary exposure, environment, or culture is unclear. The following section summarizes general patterns related to age, sex, genetics, BMI, Race/Ethnicity, and saliva, all factors that have the potential to influence nutrition through modifying food preferences.

#### Age

Sensory acuity generally declines with age, although it remains unclear whether this decline is truly a result of aging or due to increased use of multiple medications and the consequence of health disorders. Regardless of the underlying cause, the outcome is the same: older adults often have lower taste and smell acuity than younger adults [[206], [207], [208], [209], [210], [211]]. Overall, the impairments with age tend to associate with poorer nutrition [212], but the poor nutrition could also be related to other age-associated factors such as decreased independence, memory, and general test performance [[212], [213], [214]].

#### Sex and gender

Females are often more sensitive to sensations than males, both in terms of how strong sensations feel as well as in the minimum concentration at which they can detect stimuli [211,[215], [216], [217], [218], [219]]. In cross-sectional data from the United States NHANES, males also have a higher prevalence of smell, but not taste, impairment than females [220]. This difference may have implications for food preferences and dietary choices between sexes, as males, on average, may experience less intensity and be less sensitive to a variety of sensations. However, cultural differences in expected gender roles also contribute strongly to differences in eating behavior and nutritional choices for men and women [111], and these social gender roles are difficult to separate from the influence of biological sex on fundamental sensory function.

#### Genetics

Genetic factors play a significant role in individual variability in taste and smell perception [200,201]. In general, genes that contribute to greater sensitivity to various tastes or aromas may contribute to better nutritional outcomes, yet the details are much more nuanced. A genetic influence on sweet-taste receptors has been correlated with reduced sensitivity to sweetness, and then sometimes associated with higher sugar intake [[221], [222], [223], [224], [225]], but others have found no such dietary association with sweet-taste receptor genes [216]. Copy number variation (CNV) in the salivary amylase gene has been associated with obesity and higher BMI in several studies [[226], [227], [228], [229], [230], [231]]. However, it remains unclear whether this association is due to differences in textural preferences or if the CNV induces differences in how carbohydrates are digested, potentially influencing obesity. Several studies have reported potential genetic roles in associations between reduced sensitivity to oleogustus (fat taste) and preference for and higher intake of high-fat foods [[232], [233], [234], [235], [236], [237], [238]]. For odors, genetic variation contributing to intensity, pleasantness, and aroma quality has been noted for a variety of molecules that contribute to food-related aromas such as butter, fishiness, licorice, cilantro, cinnamon, and more. A detailed review of genetics and oral sensations can be found here: [200].

A clear example of how the genetics of taste do not always logically predict health is provided by the TAS2R38 gene. This gene encodes a bitter receptor (T2R38), and genetic variation in TAS2R38 leads to relatively clear phenotypes. “Tasters” and “supertasters” from the PAV (proline-alanine-valine) haplotype of this gene experience strong bitterness from the synthetic compounds phenylthiocarbamide and 6-*n*-propylthiouracil, whereas “nontasters” with the AVI/AVI (alanine-valine-isoleucine) diplotype experience no bitterness. Similar patterns of bitterness based on these polymorphisms have been observed for natural goitrin-type bitter molecules, common in cruciferous vegetables [[239], [240], [241], [242]]. Some studies show small effects indicating “nontasters” may consume more vegetables, particularly bitter greens, presumably because they find these vegetables less bitter [[243], [244], [245]]. From a nutritional perspective, one would expect that such a genetic mutation leading to greater acceptance of bitter green vegetables would then lead to more positive health outcomes, yet that is not the case. Where effects have been documented relating TAS2R38 genotype to health, the “nontaster” genotype or phenotype is typically associated with the more “negative” health outcomes, such as increased obesity/overweight, diabetes, higher preference for dietary fat, higher energy intake, and related outcomes [200,[246], [247], [248], [249], [250], [251], [252]]. Thus, TAS2R38 is an example of how the genetics of sensory variability does not always directly explain health outcomes through food choice.

#### Race and ethnicity

Ethnic variations in genetics related to chemosensory perception have been observed, and cultural differences among ethnic groups would contribute to differences in preferred sensory experiences. Some studies have reported lower scores on olfactory tests in people who identify as Black compared with those identifying as White, particularly in younger individuals [253]. Additionally, a nationwide representative sample of the United States population found differences in the prevalence and risk factors of smell impairment across ethnic groups, with minority groups displaying greater rates of impairment and non-Hispanic Black Americans showing a higher prevalence of taste impairment [220].

#### BMI

The relationship between BMI and sensory perception is complex and often contradictory. Some studies report lower sensory sensitivity or intensity perception in individuals with overweight or obesity [[254], [255], [256], [257], [258], [259], [260], [261], [262]], whereas others find higher sensitivity [[262], [263], [264]], or no effect [265]. Olfactory dysfunction has been correlated with higher BMI in United States cross-sectional data and in a meta-analysis [257,266]. Thus, a direct relationship between BMI and sensation is not clear.

#### Summary

The relationship between sensory perception and nutrition is complex and multifaceted. Although clear associations exist between sensory dysfunction and poor nutritional outcomes, the impact of normal sensory variability on diet quality and health is less straightforward. Factors such as age, sex, genetics, BMI, and ethnicity all could contribute to individual differences in taste and smell perception and valence. Presumably, these differences could then, in turn, influence food choices and nutritional status. However, the cultural influences on acceptance of sensations and food choice are difficult to fully decouple from these individual factors. Considering sensory function in terms of precision nutrition, key issues still need to be resolved. Although myriad examples of variability in sensory experience have been documented, the links between the variability and diet quality are less clear. Generally, lesser or impaired sensitivity to sensations may be associated with poorer health outcomes, but variability within normal ranges may have less strong or less consistent influences on health.

### Dietary intake

#### Search and strategy

This section first provides a context regarding the assessment and measurement error inherent to quantifying dietary intake. Next, the age, sex, and life-stage driven dietary intake needs are also reviewed with comments to the related genetic factors. A summary regarding adherence to dietary intake guidance by age, sex, and life stage is outlined based on the most recent nationally representative evaluation of current intakes. Finally, a search within PubMed was completed with terms of dietary quality, dietary intake, United States, adult children, age sex, race/ethnicity, and socioeconomic status to identify studies that evaluated dietary quality within the same dataset regarding the magnitude of dietary differences related to age, sex, race/ethnicity, and socioeconomic factors.

#### Key methods of measurement/observation

Identifying usual dietary intake and quality over the long term is a goal for dietary assessment because nutrient adequacy and dietary recommendations are intended to be met over time [267]. Dietary intake assessment methods traditionally rely on the self-report of participants [268]. This may take the form of detailed reporting of all foods and their amounts consumed in the past 24 h as in a 24-h dietary recall. Alternatively, the nature, frequency, and quantity of foods/food groups consumed over a longer reference period, for example, days, months, or years, may be reported on a questionnaire such as a food frequency questionnaire [268]. Although these 2 formats reflect past intake, the other major type of dietary assessment, the food record, requires individuals to record what is consumed as it is happening or immediately after intake, and is usually carried out over multiple days [268]. All methods rely on the complete reporting of all foods and amounts for accurate assessment. Newer methods of dietary assessment generally still rely on self-report and may include capturing images of foods using mobile applications or digital prompts to record what is being consumed at certain times of the day [269]. Other methods are more passive, such as sensor-detected video of dietary intake, yet even these may be manipulated by participants as to whether all eating events are captured. Measurement error is inherent to all methods because individuals often forget to report certain foods and beverages and misestimate the amounts. Misidentification of food by the individual, investigator, or database may also contribute to error as nutrient estimates within databases are generalized and may not account for variations in season, ripeness, storage, preparation, and brand [270]. Altered eating patterns in response to reporting burden or purposeful misreporting due to an individual’s perceptions of researcher judgment or stigma are problematic and prevalent as well.

Therefore, error exists between individuals and within an individual. In addition, the various components of dietary intake may be unequally impacted by measurement error. Energy is derived from most foods and beverages, and errors may reach 30%, like estimates for protein, with variation by sex and body size. In particular, the factors of interest in this review including age, sex, life-stage, BMI, race/ethnicity, and socioeconomic status, are all potentially related to variable measurement error. In contrast, other dietary components may be consumed episodically, so capture of the specific foods comprising those nutrients is critical for their estimation, and when appropriately measured, error may be improved, for example, potassium at ≈5% [[269], [270], [271], [272], [273], [274]]. Several methods have been designed to statistically adjust for the within-person measurement error and account for the episodic or ubiquitous intake of dietary components, yet between-person error still exists. Thus, capturing multiple days using less biased dietary assessments, that is, the 24-h dietary recall, is advised [270,275].

#### Introduction

Dietary intake, specifically the intake of foods and beverages (not including dietary supplements), is necessary not only to sustain life but also for healthy growth, disease prevention, recovery from injury and sickness, reproduction, and functionality throughout the lifespan from infancy to older adulthood [276]. Humans choose from an array of foods and beverages with varying amounts of energy, ≤3 essential macronutrients, and >20 micronutrients, plus fiber, water, and other dietary components. Too little or too much of certain dietary components may hinder health and performance [277]. The balance of dietary composition is also important as some nutrients may limit or augment the absorption and metabolism of other nutrients, leading to compromised or augmented health. The optimal balance is conceptualized and operationalized through the grouping of foods that are similar by their composition, source, or culturally determined use patterns [278]. Food groups are important for research, education, and dietary guidance on healthful selection and in determining how well the set of foods consumed aligns with needs and long-term health [279,280]. Therefore, the amount of dietary intake as energy is critical to health as is the dietary quality or variety and balance of different types of foods supplying the array of essential macro and micronutrients to achieve adequacy, with potentially unlimited ways that healthful diets may be constructed.

Dietary intake is influenced by biological needs where age, sex, life-stage, body size, and genetics may have special relevance and where intakes within a varying range are acceptable [269,272]. Social or cultural factors and the behaviors of groups and individuals may also influence dietary intake. Indicators of these sociocultural factors that are most ubiquitously studied are race/ethnicity and socioeconomic status. Each may then interact with age, sex, and life stage [[281], [282], [283]]. Yet, life and growth may be sustained for many years at less-than-ideal intake. Detrimental effects may take years to manifest with varying degrees of influence and interaction from the factors mentioned, hampering specification of their effects [277]. For example, obesity can result over years from excess energy intake, low resources to achieve dietary quality, genetics, and life-stage changes [284]. Recognition of individual autonomy to modify certain intake behaviors and preferences, the strong role of society in race/ethnicity and socioeconomic influence, and less modifiable aspects of age and genetics can inform interventions for precision health.

#### Age, sex, life-stage, body size (BMI), and genetic factors

Dietary intake needs are established for certain dietary components by sex and life stage, for example, iron for adolescent girls, folate in pregnancy [276,277,279,[281], [282], [283]]. The United States Dietary Guidelines outlines food group intake by energy level for age-sex-life-stage groups based on a compilation of scientific evidence [279]. The dietary reference intakes are evidence-based markers of requirements or of minimum levels of adequacy for energy, macro-, micronutrients, and other dietary components and upper levels of tolerance (except for energy) based on age-sex-life-stage (pregnancy and lactation) [277,285]. Body size (e.g., BMI) and physical activity level are acknowledged to also differentiate dietary intake needs for energy and certain nutrients, yet recommendations tailored to these differences are generally not outlined except in the case of energy [286]. Genetic factors such as individual variations in nutrient metabolism may manifest in a multitude of ways, that is, nutrient absorption, transport, storage, excretion, etc., and may drive individual biological needs and potentially dietary intake. Certain dietary patterns may also have a genetic link [287]. Yet in practice, these genetic factors are often unknown without specific genetic or metabolic testing, and their large-scale impacts on dietary intake and interaction with other important factors in the population are also less known or poorly understood [249,276,286]. Because needs vary based on age, sex, and life-stage, direct comparisons of intake may not be as useful as consideration of how well groups identified by these factors adhere to guidance on dietary quality or nutrient recommendations [279,280]. As a population, overwhelming clear and consistent evidence shows the quality of dietary intake in the United States, or adherence to the Dietary Guidelines for Americans (DGA), is poor, at 58 on a 100-point scale [279,280,288].

Dietary quality is lowest in adolescence with relatively small, yet steady improvements going back to infancy and similarly small, yet steady increases from adolescence to adulthood and older adulthood. Therefore, the youngest and oldest age groups generally have the highest dietary quality, though both remain low in absolute ratings. Smaller and less meaningful differences in dietary quality are apparent among groups by sex. Slightly higher dietary quality is observed for females compared with males and for life stage, as pregnant and lactating females generally have higher dietary quality compared with those not pregnant or lactating [280,288,289]. The adequacy of nutrients and dietary components varies considerably over the lifespan, yet the trend follows dietary quality, with nutrient adequacy attainment better at the youngest and oldest ages and poorest in adolescence, especially for girls who exhibit a constellation of nutrient gaps within a lower calorie allowance to accommodate requirements compared with boys [272].

#### Society and culture (including race/ethnicity and socioeconomic factors), and behavior

**FIGURE 2:**
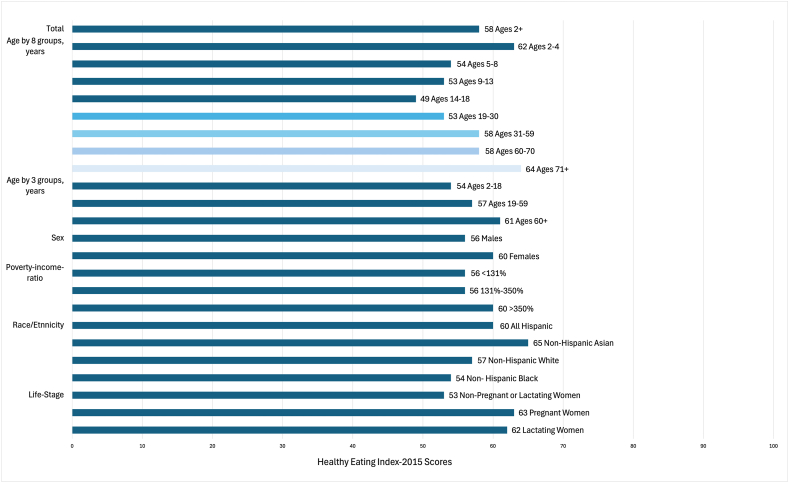
Dietary quality by Healthy Eating Index–2015 scores for Americans 2+ y from the What We Eat In America, National Health and Nutrition Examination Survey 2017–2018. ∗Note: data shown here are from the U.S. Department of Agriculture, Food and Nutrition Service, Center for Nutrition Policy and Promotion. 2021. Average Healthy Eating Index-2015 scores for Americans by Race/Ethnicity, Ages 2 Years and Older. What We Eat in America, NHANES 2017–2018 and U.S. Department of Agriculture, Food and Nutrition Service, Center for Nutrition Policy and Promotion. Average Healthy Eating Index-2015 scores for Non-Pregnant Non-Lactating, Pregnant, and Lactating Women 20–44 Years. 2021. What We Eat in America, NHANES 2013–2018.

Social, cultural, and environmental factors, and dietary behaviors and preferences may also influence intake. Because social and cultural norms and expectations are different based on age, sex, and life stage, these aspects are also relevant to dietary behaviors and intake. Individual preferences and choices can be limited or expansive based on income level, food preparation skills and facilities, cultural background, food availability, the power structures of their communities and organizations, and other factors along with the intersectionality of these aspects [[281], [282], [283], [284],^290^]. With acknowledgment of these many influences, most evidence of the comparative relationships of such factors to dietary intake is limited to basic demographic indicators such as race/ethnic group, socioeconomic status, sex, and age. Nationally representative data from the NHANES [291], provide an ideal evaluation of these basic demographic factors and mean differences in dietary quality within the same sample [288,289,[292], [293], [294], [295]]. The results are similar to those described above for sex in that factors of race/ethnicity and socioeconomic status are related to dietary quality differences, but these are small in comparison to overwhelmingly poor dietary quality and nutrient adequacy in all groups [288,289,[292], [293], [294], [295]], Figure 2 [288,289]. A recent evaluation used NHANES 2017–2018 for ages 2+ and showed mean dietary quality scores were highest among the group identifying as non-Hispanic Asian, followed by Hispanic and non-Hispanic White and non-Hispanic Black; the 2 groups reporting income <131% of the poverty-income-ratio and between 131% and 350% had lower dietary quality than those with incomes <350% [288]. Hiza et al. used earlier data from adults in the 2003–2004 NHANES and similarly showed females had more healthful overall diets than males using the Healthy Eating Index-2005. Those with Hispanic race-ethnicity had higher quality diets than non-Hispanic Black and White subgroups, and dietary quality improved with income [292]. Other evaluations of nutrients and dietary components using 2003–2008 and 2007–2010 adult data showed mixed results among sex, race/ethnic, and socioeconomic groups, where a greater number of differences were noted by race/ethnicity than by sex or by socioeconomic group, but again, all groups had poor intake overall [293,294]. Another NHANES study using 1999–2012 data compared the percentage of adults with poor diets per a measure of dietary quality and found higher intakes of healthful food groups for those with higher compared with lower socioeconomic status and for the non-Hispanic White group compared with the non-Hispanic Black or Mexican American group, yet differences were small [295]. A consistent finding in all the studies was that dietary quality was consistently poor in all groups, with low adherence to dietary guidelines and recommendations.

The evidence for children, in comparison with adults, is more limited with regards to evaluation of the relationship of sex, race/ethnicity, and socioeconomic status to dietary intake within the same study [292,293]. Yet there appears to be little difference between female and male children in dietary quality. Similar to adults, children often have lower dietary quality with lower socioeconomic status [292,293]. Few studies have gone beyond evaluating > 2 or 3 categories of each of these groups despite the wide diversity within each one. Furthermore, because race/ethnicity and socioeconomic status are mostly societal, with more limited biological constructs, the heterogeneity within these groups may be similar, if not greater than, between these groups [281,296,297]. For example, dietary quality scores were ≈11 points different between the broadest groups of non-Hispanic Asian individuals, decreasing to Hispanic, non-Hispanic White and non-Hispanic Black individuals (Figure 2) and ≈15 points different between those identifying as Mexican and as Puerto Rican in a study representing those of Hispanic heritage where Dominican, Central American, Cuban, and South American were also included [298]. The potential differences of sexual orientation to dietary quality similarly deserve more attention in the literature; 1 study found that gay or bisexual males had ≈4 points higher dietary quality compared with heterosexual males using NHANES 2011–2016 whereas lesbian/bisexual females did not differ from their heterosexual counterparts [299]. The relative differences and magnitude of those differences for each of these determinants of dietary quality and intake depend on the specific group of interest, diversity, and intersectionality of factors within that group.

#### Summary

Dietary intake plays a significant role in the risk of chronic disease, long-term and immediate health. Considering the high rates of obesity, cardiovascular disease, cancer, diabetes, osteoporosis, and other metabolic disorders, it may not be surprising that dietary quality and nutrient adequacy throughout the lifespan and among all groups by age, sex, life-stage, race/ethnicity, and socioeconomic status are poor overall. However, some groups experience disparities in dietary intake and outcomes by these indicators because of their link to social treatment, power structures, and unequitable experiences. Therefore, they underscore the need for resources and interventions to optimize equity of health for every individual [[300], [301], [302], [303], [304]].

### GI function/gut–brain axis

#### Search and strategy

To review the literature for this section, PubMed searches for gastrointestinal/satiety hormones and age, sex, genetics, BMI, and ethnicity were conducted. The following focuses on the gastrointestinal hormones most studied in relation to the regulation of dietary intake. Original research articles are cited as well as review articles that summarize findings for which a consensus has been reached.

#### Introduction

Information about the amount and composition of ingested food in the gastrointestinal tract (gut) is communicated to the brain via vagal and spinal afferent pathways. These afferent pathways receive information from the enteric nervous system, a network of neurons within the gastrointestinal tract that signals locally (and can act independently of the central nervous system) to regulate food transit, nutrient absorption, immunological responses, and blood flow [305]. Enteroendocrine cells (EEC) detect different chemical moieties, products of digestion, and microbial metabolites within the gut and produce hormones that communicate with the brain either via the vagal and spinal afferents or through the circulation [306]. This section focuses on the function of gut hormones to communicate with the brain to regulate dietary intake.

EEC in the gastrointestinal tract produce ∼20 bioactive hormones [307]. Several of these, illustrated in Table 1, have been well studied for effects on appetitive sensations. In addition, the pancreatic hormones insulin, glucagon, and pancreatic polypeptide (PP) also regulate appetite. How to interpret data on gut hormones requires consideration of several important points. First, in humans, gut hormones are usually measured in peripheral venous blood. Thus, concentrations of these factors are different (usually much lower) than what would be present in the portal circulation and sensed by receptors signaling via vagal afferents. Second, gut and pancreatic hormones can be measured in the fasting state or in response to a nutrient challenge. It is not clear that fasting gut hormone concentrations influence dietary intake to the same extent, if at all, compared with the dynamic change in these hormones following dietary intake. Third, blood concentrations of the gut hormones do not change in isolation. Rather, when food is ingested, most gut hormones increase, and ghrelin decreases. Thus, it is the combined effect of all changes that may influence dietary intake. Finally, although the infusion of gut hormones at pharmacologic concentrations has undisputed effects on appetite and dietary intake, the evidence is mixed that meal-induced changes in gut and pancreatic hormones are large enough to significantly influence appetite [308].

**TABLE 1:** Major gut and pancreatic hormones regulating dietary intake.

Hormone	Source	Effect on dietary intake
CCK	I cells in duodenum and jejunum	Suppresses hunger, promotes digestion by stimulating bile and enzyme release
Ghrelin	A (X-like) cells in fundus of stomach	Increases hunger, stimulates dietary intake
GIP	K cells in duodenum and proximal jejunum	Increases insulin secretion, may regulate appetite, and inhibit stomach acid release
Glucagon	α cells in pancreatic islets	Increases blood glucose, decreases dietary intake, promotes satiety
GLP-1	L cells in the distal small intestine and the colon	Suppresses hunger, enhances insulin secretion, and slows gastric emptying
Insulin	β cells in pancreatic islets	Decreases blood glucose, promotes satiety
PP	PP cells in pancreatic islets	Suppresses hunger, regulates pancreatic secretion
PYY	L cells in the intestine	Suppresses hunger, delays gastric emptying

**Abbreviations:** CCK, cholecystokinin; GIP, glucose-dependent insulinotropic polypeptide; GLP-1, glucagon-like peptide-1; PP, pancreatic polypeptide; PYY, peptide YY.

#### Age

GI and pancreatic hormones are detectable in blood in all age groups studied. Three-month-old formula-fed infants have higher fasting levels (3 h fast) of ghrelin, insulin, glucose-dependent insulinotropic polypeptide (GIP), PP, and lower concentrations of peptide YY (PYY) compared with breastfed infants [309]. In a subsequent report on this cohort, fasting ghrelin increased, and PYY decreased, from 3 to 6 mo of age [310]. Introduction of solid foods at 6 mo results in greater ghrelin concentrations compared with milk/formula-fed infants. Ghrelin decreased 10 min post intravenous glucose infusion in 1-y-old infants [311], GLP-1 increased in 7-d-old neonates with milk feeding [312]. Taken together, all these studies suggest the gut hormone response to dietary intake is present at an early age and is reflective of the amount of nutrients ingested.

Studies in adolescents (7–17 y) and adults (21–65 y) generally show that orexigenic hormones increase in response to meal tests or oral glucose, that ghrelin decreases with energy intake, and that there is little effect of age that can be isolated from the onset of age-related comorbidities such as diabetes (reviewed in [313]). However, older adults exhibit lower hunger and greater postprandial fullness compared with younger adults, a phenomenon termed the anorexia of aging [314]. Implicating gut hormones in the anorexia of aging, a meta-analysis indicated older adults (mean age 73 y) exhibited higher fasting and postprandial concentrations of cholecystokinin (CCK), PYY, and insulin compared with younger adults (28 y), with no significant differences in GLP-1, GIP, or ghrelin [315]. A 4-criterion classification scheme [316] was used to separate older adults into those with a “healthy appetite” compared with those with “suppressed appetite.” These investigators reported greater postprandial suppression of ghrelin with larger increases in postprandial GLP-1 and PYY in “appetite-suppressed” older adults compared with young adults. These alterations in gut hormone response to meal intake were not observed in the older adults with “healthy appetite.” It will be important to determine the mechanism(s) driving these observations.

Older adults exhibit modestly slower gastrointestinal motility, but gastric emptying rates remain within the range of those in young adults. Thus, significant effects on appetite are unlikely. However, disease states with greater prevalence in older adults, such as type 2 diabetes mellitus and Parkinson’s disease, can be associated with significant gastroparesis resulting in reduced appetite [317].

#### Sex

An effect of biological sex to influence fasting or postprandial gut hormone concentrations in healthy humans is controversial, with some studies suggesting higher concentrations in females and other studies finding no difference or lower concentrations (reviewed in [314]). One confounding factor in these analyses is the effect of adiposity on postprandial hormone release (discussed in detail below), with most studies matching males and females by BMI, but not fat mass or fat distribution. The menstrual cycle may also impact gut hormone responses. Two studies in small groups of females suggest that the PYY response to meal intake is less in the luteal compared with the follicular phase [318] or, in contrast, that glucose-induced GLP-1 is higher in the follicular phase of the cycle with no difference in CCK [319]. It is not clear if menstrual cycle-related differences would be detected with larger study groups.

Although gut hormone concentrations may not differ between males and females, there is good evidence from ovariectomized rodents that estrogens enhance the anorexigenic effects of CCK and GLP-1 [320]. There is also evidence from mouse and human intestinal explants that estrogen activates estrogen receptor β (ERβ) on L cells to increase GLP-1 release [321]. A direct effect of estrogen to increase GLP-1 secretion from EEC could influence appetitive neural pathways originating in the vagus nerve and have a minimal effect on circulating GLP-1 concentrations.

#### Genetics

Data linking genetics to altered gut hormone release are limited. Variants in *FTO* locus are associated with increased body weight and energy intake. A recent study reported no association of the rs9939609 genotype with fasting or postprandial GLP-1 or PYY [322]. Interestingly, postprandial ghrelin was increased in females with genotype AA and increased fat mass compared with genotypes TT and AT. Polymorphisms in the CCK and the GLP-1 receptor are associated with hyperphagia and risk of overweight/obesity [323].

#### BMI

Excess adiposity is associated with significant changes in circulating pancreatic and gut hormones compared with concentrations in individuals with a healthy weight. Fasting and postprandial insulin are elevated in individuals with obesity and insulin resistance (see [324] for one of many studies demonstrating this). Glucagon, which should be suppressed in the presence of hyperinsulinemia, is also elevated in insulin-resistant individuals with obesity [325], contributing to the fasting and postprandial hyperglycemia in these individuals. Although both insulin and glucagon are anorexigenic in infusion studies, the elevated levels of these hormones in obesity have little effect on appetite.

Fasting PYY concentrations are inversely associated with BMI and are thus lower in people with obesity [326]. This study also showed that the postprandial rise in PYY is attenuated (resulting in a smaller area under the curve) in people with obesity despite their consumption of more energy during a buffet meal test. An attenuated postprandial rise in PYY in people with obesity has been observed in additional studies [327,328].

There is no consensus that fasting GLP-1 concentrations are altered in obesity, although the postprandial increase in GLP-1 is attenuated as observed for PYY (reviewed in [329]). A recent study observed that suppression of glucose-induced postprandial GLP-1 occurred in adolescents with obesity and insulin resistance, but not in adolescents with obesity who were insulin sensitive [330].

Fasting ghrelin is inversely associated with BMI and body fat [331], and is reduced in people with obesity. Circulating ghrelin concentrations rise in the fasting state and fall following meal intake in people with obesity, but the diurnal profile is always lower than that in people with a healthy weight [332,333]. In addition, some studies find that people with obesity exhibit less suppression of ghrelin with meal intake [328]. It has been suggested that elevated insulin in the obese state suppresses ghrelin [334,335]. Although ghrelin is often thought of as a hunger hormone driving meal initiation, interesting work in rodent models suggests that the rise in ghrelin might be more to prepare the body for incoming food to metabolize and store energy efficiently [336].

It is difficult to establish cause-and-effect for the change in gut hormone secretory patterns in obesity. A recent meta-analysis indicated that fasting ghrelin increased, but PYY and GLP-1 decreased, with weight loss achieved by energy restriction, exercise, or the combination of energy restriction and exercise [337]. Other studies find that postprandial increases in GLP-1 and PYY are attenuated in people who have lost ≥8% of body weight, supporting the view that reduced anorexigenic hormone release contributes to greater appetite and weight regain with dieting [329]. In contrast, postprandial GLP-1 and PYY are significantly increased following bariatric procedures [338]. This increase is attributable in part to the more rapid entry of nutrients into the distal gut. Interestingly, the number of EEC in the gastrointestinal tract is reduced in people with obesity, and increases following bariatric surgery [323].

#### Race and ethnicity

Data linking ethnicity to altered gut hormone release are limited. In one study comparing middle-aged overweight Black and White females, fasting and postprandial PYY were lower in the Black females [339]. A similar suppression of postprandial PYY was noted in Black females with obesity [340]. Suppression of ghrelin in response to a low glycemic test meal was less in Black females (test group included 10 normal weight and 10 people with obesity) compared with a similarly balanced group of White females [199]. An attenuated postprandial PYY increase, and ghrelin suppression was also observed in Black adolescents and middle-aged adults with a range of BMI from healthy weight to obesity in 2 separate studies [341]. An important consideration in these studies is that adipose tissue amount and distribution differ between Black and White people, and adiposity itself alters gut hormone concentrations.

#### Summary

The gastrointestinal tract and pancreatic hormones signal to the brain the type and amount of nutrients ingested. These signals reach the brain via neural circuits and the circulatory system. Obesity results in lower concentrations of GLP-1, PYY, and ghrelin, whereas insulin and glucagon are increased. It is not clear if the change in gut hormones with excess adiposity is a cause or consequence of obesity. Advanced age lowers gut hormones compared with younger adults, and there are some effects of biological sex, race, and genetics. There is no consensus that the variation in the physiological concentrations of gut hormones has a significant effect on appetite or regulation of weight gain. However, the development of pharmacologic therapies based on GLP-1 has revolutionized the ability to induce medically significant weight loss [342].

### Gut microbiome

#### Search and strategy

This narrative review focuses on the impacts of different factors on the gut microbiome, ingestive behavior, and energy intake. These factors included genetics, BMI, race/ethnicity, age, and sex. We conducted a PubMed and Google Scholar search with keywords such as “gut microbiota” or “gut microbiome,” with “genetics,” “age,” “sex,” “race,” “ethnicity,” and “energy intake.”

#### Key methods of measurement/observation

Overall, human studies that include the gut microbiota or microbiome as an outcome have primarily focused on DNA extracted from fecal samples, followed by amplicon sequencing or metagenomics. Amplicon sequencing often amplifies the 16S rRNA gene—a highly conserved gene in bacteria and archaea—that contains 9 hypervariable regions [343]. Primers targeting one or several regions of the 16S rRNA gene are often used. For example, the Human Microbiome Project utilized primers targeting the V1 to V3 or V3 to V5 regions, whereas the Earth Microbiome Project targeted the V4 region of the gene [344,345]. These sequences are read in next-generation sequencing platforms, and through downstream analyses, these sequences are aligned to phylogenetic trees and matched to available databases, providing broad metrics of diversity and relative composition, often up to the genus level [346,347]. Although the cost of amplicon sequencing has been decreasing with fast technology advancements, the main limitations include resolution up to the genus level and data focused on bacteria and archaea. On the other hand, shotgun metagenomics fragments the genomic DNA in a sample (without targeting a specific gene), sequences those fragments, and then downstream analyses assemble those fragments and map them into available databases, also providing metrics of diversity and composition up to the species and strain levels, and potential function [346,347]. In contrast with amplicon sequencing, shotgun metagenomics can evaluate all microorganisms, not only bacteria and archaea, but relies on more substantial computational and bioinformatic capacity. In addition to amplicon sequencing and metagenomics, other methods are also possible, but less prevalent in the available literature, and these include meta-transcriptomics and meta-proteomics [347].

#### Introduction

The human microbiome is a multispecies assemblage of living organisms present in various body sites and includes the structural elements that make up these microbes and the metabolites they form [348]. Microbial cells outnumber their human hosts at a 1.3:1 ratio, whereas microbial genes outnumber human genes 150-fold [349]. As such, the human microbiome constitutes a highly abundant and metabolically active organ that may be influenced by environmental factors including diet. Given the abundance of microbial cells and the proximity of unabsorbed dietary components in the colonic lumen, the vast majority of human microbiome research has focused on the gut microbiome [350]. Indeed, early work demonstrated that microbial composition and the production of physiologically relevant metabolites could be changed by diet in as little as 1 d [351]. Furthermore, taxonomic diversity and activity of the microbiome are highly personalized and influenced by external factors including age, biological sex, race, geography, mode of delivery, and diet [352]. In the next section, we review these factors in greater detail and their influence on the microbiome, ingestive behavior, and energy intake.

#### Age

During early years of life, particularly the first 1000 d, the gut microbiome undergoes rapid and significant changes. This period is characterized by the establishment, rapid reorganization, and stabilization of the microbial community. The preliminary microbial colonization of the infant gut is largely dependent on mother-infant trade-offs of microbiota [[353], [354], [355]]. A cluster of factors and practices, including mode of birth, perinatal and postnatal antibiotics usage, feeding method, and environmental exposures, has been established to affect the assemblage of the infant gut microbiome [[355], [356], [357]]. In the first week of life, the infant microbiome is colonized by facultative anaerobes like *Streptococcus* and unclassified Enterobacteriaceae, which are thought to reduce the gastrointestinal environment, allowing for the establishment of anaerobic microbes, including *Bacteroides* and *Bifidobacterium* species [358,359]. The microbial structure becomes further complex and diverse as food (i.e., breastmilk, solid food) is introduced [360]. It was previously suspected that the human gut microbiome becomes stable after 3 y. However, current evidence suggests that it remains dynamic and susceptible to changes compared with later stages of life, as demonstrated among children aged 7–12 y [361].

During adulthood, the gut microbiome reaches a relatively stable state and is typically dominated by two major phyla: Firmicutes and Bacteroidetes, with other phyla such as Actinobacteria and Proteobacteria present in smaller proportions [362]. This stability is important for maintaining various bodily functions, including digestion, immune response, and metabolic processes. However, stability does not imply rigidity. The adult microbiome can be influenced by modifiable factors including diet, lifestyle, and antibiotics leading to changes in composition that are associated with health outcomes. Moreover, the gut microbiome's stability during adulthood provides a baseline against which age-related changes in older adults can be compared [363].

As individuals enter their senior years, the gut microbiome undergoes gradual but significant compositional shifts that are associated with declining health and increased frailty. In older adults (65+ y), core genera such as *Bacteroides, Alistipes*, and *Parabacteroides* increase in abundance [363]. Interestingly, centenarians often exhibit higher gut microbial α-diversity compared with younger elderly individuals, which is thought to contribute to better health outcomes and longevity [364,365]. In particular, the loss of core taxa such as *Bacteroides, Roseburia*, and *Faecalibacterium*, along with an increase in rare taxa, has been noted [366,367]. This suggests that the gut microbiome continues to evolve and adapt even in the later stages of life, potentially reflecting the body's changing physiological state and immune function. Overall, although there appear to be periods of dynamism and stability on the gut microbiome related to stages of the life cycle, these life stages are also strongly influenced by a variety of factors, including environmental factors, which may enhance the variability of the gut microbiome.

#### Sex

Extensive crosstalk between human sex hormones and resident microbes impacts sex steroid reabsorption, gastrointestinal physiology, and gut microbial composition. Sex steroid hormones are synthesized primarily in the ovaries, testes, adrenal glands, and adipose tissue and share a common steroid backbone derived from cholesterol. Endogenous estrogens and androgens are conjugated in the liver and subject to biliary excretion [368]. Colonic exposure to sex steroid hormones differs by biologic sex and menopausal status and varies throughout the menstrual cycle in healthy premenopausal females [369]. Furthermore, microbial transformations of fecal steroids by the sterolbiome can alter sex steroid hormone potency, systemic half-life, and receptor affinity [368,370]. For example, colonic concentrations of sex steroids may be increased via the microbial conversion of glucocorticoids to androgens [370,371]. Additionally, cleavage of conjugated sex steroids by sulfatase and β-glucuronidase-harboring microbes allows for colonic reabsorption, thereby increasing systemic concentrations [368,370,372]. Consequently, compared with males and postmenopausal females, premenopausal females have an enrichment of microbial steroid degradation and biosynthesis pathways that are associated with serum levels of testosterone and progesterone [372].

Colonic exposure to sex hormones appears to have differential impacts on gastrointestinal motility and barrier function. Slower gastrointestinal transit is observed in premenopausal females compared with both males and postmenopausal females not taking hormone replacement therapy [373]. In male Swiss CD1 mice, androgen receptor activation by dihydrotestosterone triggers a signaling cascade that induces calcium sensitization of intestinal smooth muscle, thereby increasing contractile activity [374]. Conversely, binding of estrogen to the nuclear ERβ activates the large conductance calcium-activated potassium channel, thereby inhibiting smooth muscle contractile activity in the colon of rats [375]. Furthermore, estrogen treatment increases mucin production, and deletion of Erβ leads to disorganized mucin localization in intestinal epithelial cells. Accordingly, the mucin-degrading species *Akkermansia muciniphila* has been consistently observed to be significantly more abundant in microbiomes of females with normal BMI [[376], [377], [378], [379], [380]]. Thus, differences in gastrointestinal transit and mucin production likely lead to differences in luminal substrate availability and pH that drive sex steroid hormone-related differences in microbial composition [368].

Results from studies examining microbiome diversity and taxonomic composition have been inconsistent, but some trends have emerged. In one of the first studies to account for menopausal status when examining sex differences in microbiome composition, premenopausal females had an increased abundance of β-glucuronidase-harboring genera, including *Clostridium, Alistipes, and Ruminococcus,* compared with males and postmenopausal females. Notably, sex and menopausal status differences were not observed in participants with obesity. Consistent with this, urinary estrogen metabolites have been observed to be associated with fecal unclassified Ruminococcacae in two studies including *Ruminococcus*, *Oscillibacter,* and *Subdoligranulum* [381,382]*.* Factors that influence colonic sex steroid concentrations and sex associated microbiome differences are multifactorial and include biologic sex, menopausal status, obesity, use of hormone replacement therapy, timing within the estrous cycle, and conditions that may alter systemic sex hormone concentrations like polycystic ovarian syndrome [369,376,[383], [384], [385], [386]]. Furthermore, the impacts of oral contraceptive use and gender-affirming therapies on gut microbiome-sex steroid hormone interactions are still emerging [387,388]. This is further complicated by differential consumption of dietary phytoestrogens whose structural similarity to endogenous estrogens allows binding to the endoplasmic reticulum, which may have downstream impacts on gastrointestinal physiology [47,389]. Due to these complex interactions, more work that examines microbial functional genes, sex steroid hormones, and their metabolites in diverse cohorts is needed to observe consistent trends in microbial composition that may be impacted by diet.

#### Genetics

There have been several factors supporting the impact of host genetics on the gut microbiome, including dietary preferences driving intake, luminal contents and physicochemical properties, immunity, and other factors [390]. Seminal twin studies have shown that there is heritability of specific gut microbes. In several of these studies, the ACE model is often used to answer the contribution of genetics to the gut microbiome [391]. This model evaluates the variability explained by heritability/genetics (A), the contribution of the common environment between samples (C), and the unique environment effects (E) [391]. In 250 adult female twins from the Twins UK registry, using shotgun metagenomics, the similarity between the gut microbiome was higher in monozygotic compared with dizygotic twins, a result that was previously reported using 16S rRNA gene amplicon sequencing [[391], [392], [393]]. *Dorea* was one of the 11 genera displaying possible heritability, suggesting that ∼40% of the genus variability could be explained by genetics. However, important to note was the 95% confidence interval (CI) for several of these 11 genera. In the case of *Dorea*, the 95% CI was 0.094, 0.583, meaning that the explained variability could fall between 9% to almost 60%. However, for most of the other genera, the lower end of the CI was close to 0%.

Another area of interest is the possible heritability of gut microbiome components driving specific phenotypes, such as obesity. To exemplify this concept, *Blautia* has been proposed as a genus with a possible association with increased adiposity [390]. In an analysis of the Twins UK cohort [390,394], 30% of the variability in *Blautia* was explained by genetics (95% CI: 0.08, 0.37), and this genus was positively associated with CD36 and visceral fat mass. CD36 is involved in fat sensing in the tongue and the intestinal absorption of long-chain fatty acids, suggesting a possible mechanism [395]. However, as it has been the case with other genera, the data on *Blautia* and obesity are ambiguous, as some strains have been proposed to have probiotic effects. Therefore, although host genetics may impact the gut microbiome, the variability is likely high [392].

#### BMI

Although it has been suggested that BMI impacts richness (i.e., α-diversity), overall community composition (i.e., β-diversity), and drives taxonomical differences, the data are inconsistent. A recent systematic review and meta-analysis reported a nonstatistically significant difference in α-diversity using the Shannon index—an index that considers abundance and evenness of microorganisms—when 7 cross-sectional studies were meta-analyzed, pooling almost 300 individuals per group of individuals with BMI <30 compared with ≥30 kg/m^2^ [396]. In another meta-analysis of 18 studies with a range of 17–2872 participants (5–1040 with obesity) from 10 countries, a higher variance in beta diversity was attributed to the differences between studies (*R* = 0.303) rather than the difference between people with obesity and controls (*R* = 0.042) [397]. Furthermore, although a taxonomical signature in obesity has been proposed, this has been inconsistent and refuted when applied to extensive microbiome projects, including the Human Microbiome Project and MetaHIT (METAgenomics of the Human Intestinal Tract) [398].

A mechanism by which the gut microbiome may impact weight gain and body composition is through an enhanced efficiency for energy harvesting by microbes [399]. Lean germ-free mice inoculated with the microbiota of mice with obesity showed greater weight and fat mass gain compared with those inoculated with the microbiota of lean mice, despite having similar food consumption [399]. Furthermore, mice with obesity had a higher concentration of acetate and butyrate—two short-chain fatty acids—in the proximal large intestine contents but lower fecal energy compared with lean mice, suggesting that the microbiota may confer a greater energy harvest in obesity [399]. This mechanism was tested in a short-term clinical trial in people with obesity and impaired glucose tolerance, where an energy-restricted diet and using oral vancomycin administration to perturb and deplete the gut microbiome led to more energy loss in stool [400]. Another short-term crossover trial in an inpatient setting compared diets with 3400 kcal/d compared with 2400 kcal/d that lasted 3 d in individuals with BMI >18.5 and <25 compared with BMI ≥30 kg/m^2^ [401].Although the authors reported changes in the gut microbiome after 3 d, the percent of calories lost in feces between the diet treatments was highly variable (2.1%–9.2% for the 2400 kcal/d diet and 1.6%–7.6% for the 3400 kcal/d diet) with no difference reported between BMI groups [401]. Overall, although there may be an effect of BMI on the gut microbiome linked to energy harvest supported by multiple preclinical studies, the clinical data suggest high variability even in relatively tightly controlled studies.

#### Race and ethnicity

The human microbiome is distinctive to each individual and shaped by host genetics and modifiable factors including diet, early-life exposomes, lifestyle, antibiotics, and the environment [351,394,[402], [403], [404]]. Given this varied influence, ascribing microbiomes based on race and ethnicity is complex due to several factors: *1*) race is a social construct aimed to define identity based on physical attributes, *2*) geographic, social, and cultural differences based on ethnicity are often too broad to account for individual lived experience, *3*) modifiable influences to the microbiome are highly impacted by social, financial, and structural resources in the physical environment, and *4*) there is a paucity of microbiome data from diverse datasets which has truncated such observations [[405], [406], [407], [408]].

In recent years, racial and ethnic variations in gut microbial composition have been considered, though results are often tightly linked to differences in diet. In 1999, one trial demonstrated differences in microbial methane production between White and Black South African adults, which was attributed to differences in dietary intake [409]. Another trial [410] compared microbiomes among Amerindian, Malawian, and American participants across the lifespan and found distinct differences in phylogenetic composition among participants living in the United States compared with the other two countries. A comparison of functional genes demonstrated that Amerindians and Malawians who consume a maize and cassava-rich diet have a predominance of the starch-degrading gene α-amylase, whereas United States participants were enriched with metabolic genes for amino acids, simple sugars, sugar substitutes, host glycans, and bile acids [410]. Other work [409] revealed stark differences in microbial composition between South African and African-American Blacks, and in a 2-wk dietary exchange demonstrated that consuming a traditional South African Black maize-based diet increased the abundance of microbial species involved in fiber fermentation, with reciprocal changes in amino acid and bile acid metabolizing bacteria with consumption of a standard American diet [409]. More recently, a nationwide study of 2678 healthy Chinese participants from 8 ethnic groups observed that geography explained the largest variation in microbial composition and that functional prediction of the microbiome was associated with the consumption of wheat as a staple food [411]. Together, these studies demonstrate that cultural and geographical differences in staple foods are strong drivers of microbial composition that may otherwise be associated with race or ethnicity.

Racial and ethnic differences have also been investigated among participants sharing a similar geography. Using 16S rRNA gene sequencing data collected from 1673 individuals in the Human Microbiome and American Gut Projects, race and ethnicity were a stronger predictor of gut microbial composition than other covariates (sex, age, BMI) and differences in key taxa were reproducible among datasets [412]. A study of 2084 participants from six ethnic groups in Amsterdam exhibited ethnic differences in α-diversity that were independent of metabolic health, and only partly explained by dietary pattern or lifestyle behaviors. Authors noted the majority of non-Dutch participants were first-generation immigrants whose microbiomes may have been shaped by their environment before migration [413]. Indeed, although it has been shown in the United States that dietary acculturation may result in the loss of native microbial species in foreign-born participants, racial and ethnic differences in microbial composition may emerge as early as three mo of age and persist until adulthood [414,415]. In a Detroit-based birth cohort, researchers demonstrated that these early-life changes are strongly associated with mode of delivery, ingestive behaviors, household income, maternal marital status, and maternal education. Notably, many of these covariates differed by race, demonstrating that socioenvironmental barriers in early life may set the stage for health disparities in adulthood that may be related to microbial function [416].

Indeed, there is accumulating evidence that the microbiome may be a mechanism driving health disparities. It has been demonstrated that the Cheyenne and Arapaho American Indian Tribal communities had a higher percentage of individuals living below the federal poverty level and receiving supplemental assistance compared with other non-native communities in Oklahoma. Correspondingly, Cheyenne and Arapaho participants had a reduced abundance of the protective bacterial genus *Faecalibacterium* and a greater abundance of proinflammatory microbial metabolites including lithocholic acid and cadaverine compared with non-native participants [417]. In a comparison of African American, Black, and non-Hispanic White participants, Yazici and Wolf and colleagues found *Bilophila wadsworthia* to be a significant marker of colorectal cancer in African-American Blacks. Although dietary fat and cysteine intake were associated with microbial gene abundance, race remained the strongest predictor, pointing to combined dietary and socioenvironmental exposures in early life or adulthood that may shape microbial composition beyond diet [418]. This is supported by a more recent controlled dietary intervention where longitudinal multiomic profiling demonstrated persistent variation in microbiome profiles by race in Black and White female participants of similar age, weight, diet, and health status [419]. Collectively, these studies suggest that improvements to the microbiome may require interventions that reduce socioenvironmental barriers at multiple levels of influence.

#### Summary

The taxonomic diversity of the gut microbiome is dynamic throughout the lifespan due to changes in dietary intake and gut transit, which may be influenced by sex hormones. Energy harvest may be increased in individuals who have a higher BMI, though clinical evidence of this is highly variable. This is likely due to the functional redundancy of the microbiome despite highly individualized taxonomy. Thus, much work is needed to determine if personalized diets can harness the microbiome to improve health outcomes.

## Overarching Concepts

Identifying specific and tailored foods or components of an optimal diet to promote health at the level of the individual or small group is complex due to the large number of contributing inputs and the fact that many are in constant flux. Environmental or external cues may guide decision-making, constrain behaviors, and alter metabolic processes. Genetic, biological, or other internal factors will influence how organisms interact with the environment (e.g., influence chemosensory function) and determine the fate of ingested nutrients and other foodstuffs. Eating is the intermediary between external and internal environments and their signaling systems, yet this process is uniquely under volitional control. Thus, it is a critical control point in matching diets to health.

In this review, we have highlighted key drivers of ingestive behavior: culture, appetitive sensations, chemosensory function, dietary intake, the gut–brain axis, and microbiome, and their modulation by age, sex/gender, race/ethnicity, genetics, and BMI. Where evidence is available, we attempted to quantify the magnitude of the relevant factors (e.g., appetitive sensations account for <25% of the variance in dietary intake). This provides initial insights into the appropriate weighting of contributors to intake. Second, this review highlights the degree of individual variability in attributes or responses to an intervention (e.g., SNPs in the TAS2R38 gene leads to ∼25%, 50%, and 25% of individuals being super tasters, tasters, and nontasters, respectively, for selected bitter compounds). However, in many instances, interindividual variability exceeds intrasubject variability. Third, each section documents that cause-and-effect relationships are difficult to establish, as most are interactive (e.g., diet affects the microbiome and the microbiome modulates dietary intake). Fourth, there are inherent and learned contributions to both behavior and biology that will require different considerations and that offer different opportunities for manipulation (e.g., a large number of gut peptides have been identified with variable evidence that they modulate appetite, but, at the same time, their release is entrained by lifestyle and customary eating patterns). Addressing both concurrently will be required to achieve the most successful outcomes, as they are susceptible to offsetting reciprocity. Fifth, a focus on intuitive approaches may not be as successful as desired. For example, people often eat when not hungry or do not eat when they are. Some sensory qualities are unpleasant, but, in context, become desirable (e.g., bitter notes in chocolate or coffee). There is little evidence for “body wisdom” consistently or beneficially guiding food choice, especially when the effects are years in the making. Nutrient inadequacy occurs in the United States primarily because of poor food choices or options, rather than because the availability of needed nutrients is lacking. Sixth, this review highlights the point that external influences can, and often do, override internal influences of biology. Culture, health beliefs, cost, convenience, and sensory appeal can alter or overwhelm endocrine, metabolic, and neural signaling. This is not to indicate that the internal biological determinants of intake are insignificant. Instead, the point is that failure to adequately account for the important external inputs will likely compromise success in meeting preventive or therapeutic goals. To this point, a seventh observation to emerge is that, unfortunately, tools to quantify behavioral and subjective sensations are less than optimal. Difficulty in the measurement of dietary intake in free-living people is well recognized. Similar concerns can be raised about the measures of cultural practices, appetitive sensations, relevant dimensions of the microbiome, or real-time brain reward activity in free-living people. Additional attention to methodologies to capture and interpret ingestive behaviors is needed.

We propose that all these factors are critical when considering the development of advanced algorithms for precision nutrition. Yet, we also point out that no databases currently exist that contain all these types of data. We do not have large datasets that contain oral chemosensory responses and microbiome composition and appetitive sensations. Without such data, in large volumes, algorithms will end up generalizing to the largest portions of society, thus defeating the purpose of “precision” nutrition. Thus, to create true, personalized nutrition recommendations, new datasets or new methodologies to link isolated datasets are needed to allow learning or predictive algorithms to connect data across fields. Algorithms are only as good as the data they learn from, and so addressing the issue of combining data across fields and methodologies will need to occur before good precision nutrition recommendations, driven by data, can be developed. We hope that the topics covered in this narrative review will highlight the importance of these topics and their modulators, as we have discussed, so that efforts to develop algorithms can seek these data for inclusion as features in the models.

This review does not provide answers to the questions posed in the introduction, but does highlight their importance. Given the pivotal roles played by cultural norms, variability of sensory function, and how these inputs alone modify biology and contribute to food choice, coupled with the need for diets to be acceptable, not just contain an optimal nutrient profile—the answer must be that there is no single ideal diet. As noted, the diets of the Arctic Inuit and the Massai pastoralists of Kenya and Tanzania are well suited to their lifestyles and environments, but nutritionally disparate. This principle has been foundational to the last several rounds of the DGA. Indeed, the most recent version of the DGAs suggests a generalized dietary pattern that can be modified to fit previously separate omnivore, vegetarian, and Mediterranean patterns. Finally, diet, nutritional biology, and health are highly interactive. The result is not convergence on a narrowly defined homeostatic response but rather a range of responses to any input that results in marked individual differences in this balance. The boundaries of which still ensure good health are yet to be characterized. Understanding the range of acceptable outcomes will be essential to setting targets for precision nutrition.

## Author contributions

The authors’ responsibilities were as follows – RM: conceived of the review and had primary responsibility for final content; and all authors: developed the format and content of the review, wrote sections, edited the full final version, read, and approved the final manuscript.

## Funding

The authors reported no funding received for this study.

## Conflict of interest

The authors report no conflicts of interest.
